# A fitted operator numerical method for singularly perturbed Fredholm integro-differential equation with integral initial condition

**DOI:** 10.1186/s13104-023-06649-9

**Published:** 2024-01-15

**Authors:** Aklilu Fufa Oljira, Mesfin Mekuria Woldaregay

**Affiliations:** 1 Department of Mathematics, College of Natural and Computational Science, Oda Bultum University, Chiro, Ethiopia; 2 Department of Applied Mathematics, Adama Science and Technology University, Adama, Ethiopia

**Keywords:** Primary 65L10, Secondary 65D30, 65L20, 65L70, Singularly perturbed, Fredholm Integro-Differential Equation, Integral condition, Fitted operator method, Stability analysis, Uniform convergence

## Abstract

**Objectives:**

In this paper, a uniformly convergent numerical scheme is proposed for solving a singularly perturbed Fredholm integro-differential equation with an integral initial condition. The equation involves a left boundary layer which makes it difficult to solve it using the standard numerical methods. A fitted operator finite difference method is used to approximate the differential part of the equation and the composite Simpson $\frac{1}{3}$ rule is used for the integral parts of the equation and the initial condition.

**Result:**

The stability bound and error estimation of the approximated solution are performed, to show the uniform convergence of the scheme with order one in the maximum norm. Numerical test examples are provided to calculate the maximum absolute errors, thrgence, and the uniform error for a couple of examples to support theoretical analysis.

## Introduction

A wide range of mathematical models, from fluid dynamics to mathematical biology, can be expressed in the form of singularly perturbed problems. Examples of such models include high Reynold number flow in fluid dynamics and heat transport problems [21, 24]. Singularly perturbed differential equations are a class of equations frequently encountered in applied mathematics, characterized by the presence of a small positive parameter $\varepsilon$ multiplying some or all of the highest order derivative terms in the differential equations [18, 20]. These equations give rise to solutions that exhibit multi-scale phenomena [24], where certain regions of the domain feature thin layers with significant changes in scale in the solution and its derivatives. These layers are referred to as boundary or interior layers, depending on their location within the domain.

It is important to note that classical numerical methods for solving singularly perturbed problems (SPPs) are generally unsuitable for small values of the perturbation parameter $\varepsilon$, as they often lead to instability and inaccurate results. As such, appropriate numerical methods must be employed to solve these problems accurately. Due to the widespread applicability of SPPs in many scientific and engineering fields, research in this area continues to be of great importance, with ongoing efforts aimed at developing better numerical techniques for solving these types of equations. The accuracy and convergence of the methods have to be considered since the numerical treatment of SPPs is not easy and because the solution depends on the perturbation parameter $\varepsilon$ and the mesh size [20, 27].

A singularly perturbed Fredholm integro-differential equation (SPFIDE) possesses a significant barrier for numerical analysis, which demands accurate numerical results. As a result, numerous works that concentrate on designing effective numerical techniques for handling the perturbation have been published. For the purpose of getting accurate numerical results, the literature is replete with material on the numerical handling of these issues. Durmaz et al. in [7–15] considered different forms of the singularly perturbed integro-differential equations. They used different approaches to approximate the differential parts of the equations and used the composite trapezoidal rule for the integral part of the equations. Amiraliyev et al. [2] presented an initial-value problem for a singularly perturbed Fredholm integro-differential equation. They derived explicit theoretical bounds for the continuous solution and its derivative. They established parameter uniform error estimates for the approximated solution.

Hamoud et al. [16, 17] presented a variational iteration method for solving the Fredholm integro-differential equation. They provided an analytical approximation to determine the behavior of the solution. Moreover, proof of the existence and uniqueness results in the convergence of the solution. Kudu et al. [19] considered the singularly perturbed initial value problem for a first-order Volterra integro-differential equation with delay. They construct and analyze a numerical method that satisfies a uniform convergence irrespective of the perturbation parameter on the layer-adapted mesh. The numerical solution was discretized using implicit difference rules for the differential part and composite quadrature rules for an integral part. Cakir et al. [5] constructed a finite difference scheme for a first-order singularly perturbed Volterra integro-differential equation (SPVIDE) on a Bakhvalov-Shishkin mesh. They used integral identities and dealt with the emerging integral terms with interpolating quadrature rules. In their work, they established the stability bound and the error estimates of the approximate solution. In authors in [6], established a $\varepsilon$-uniform numerical scheme to solve second-order singularly perturbed Volterra Fredholm integro-differential equation. They used a non-uniform mesh by using interpolating quadrature rules and the linear basis functions. Solomon et al. [4] applied the exact difference method for solving reaction-diffusion type SPFIDE and established the uniform convergence analysis of the scheme.

Amiraliyev et al. [2] established parameter-uniform error estimates for the approximate solution of a SPFIDE. They derived theoretical bounds on the continuous solution and its derivative. A singularly perturbed second-order Fredholm integro-differential equation with a discontinuous source term was examined by [23]. They used a fitted numerical method on a Shishkin mesh to solve the problem. The method was shown to be uniformly convergent concerning the singular perturbation parameter. A singularly perturbed Fredholm integro-differential equation was considered by [3]. For the solution of the problem, a fitted difference scheme was constructed on a Shishkin mesh. The method was based on the method of integral identities with the use of exponential basis functions and interpolating quadrature rules with the weight and remainder terms in integral form. Amirali et al. [1] proposed a fitted difference scheme for first-order singularly perturbed quasilinear Fredholm integro-differential equation with integral boundary conditions using exponential basis functions, quadrature interpolation rules and the method of integral identities.

In [22], the fitted mesh finite difference method is considered for solving singularly perturbed Fredholm integro-differential equation. The derivative part is approximated using the upwind scheme and the integral part was estimated by the iterative quadrature rule. In addition, Richardson extrapolation was used and second-order accuracy was achieved. In [18] a parameter-uniform numerical method for a parameterized singularly perturbed differential equation containing integral boundary condition was studied. A numerical algorithm based on an upwind finite difference operator and a piece-wise uniform mesh is constructed. A uniform error estimate for the numerical solution was established. In [25], the authors introduced a fourth-order scheme of exponential type for solving Volterra integro-differential equations with singular perturbation. They also conduct a stability analysis of the method and provide numerical results, along with comparisons to alternative schemes.

Differential equations with integral boundary conditions will occur in many applications, for example, in heat conduction, thermo-elasticity, plasma physics, underground water flow, etc. [26]. Based on the literature review we made, it is evident that numerous numerical methods have been employed to solve first-order SPFIDEs and presented diverse findings and conclusions. Our objective in this paper is to develop an accurate and uniformly convergent numerical method for solving SPFIDE with integral initial conditions. To overcome the problem associated with classical numerical methods, we will develop the fitted operator finite difference method together with Simpson’s rule to solve the problem without generating an oscillation or divergence. Furthermore, we establish the stability and convergence analysis of the scheme. Implementation of the scheme involves the use computer program on Matlab software for numerical computation. To validate the obtained result, examples are considered and the results are presented using graphs and tables.

### Notation 1.1

The norm $\Vert .\Vert _{\infty }$ which is defined as $\Vert g\Vert _{\infty }=\underset{x\in [0,1]}{\max }|g(x)|$ is the maximum/supremum norm, for a function *g* defined on the domain [0, 1], *C* is a positive constants which is independent of the perturbation parameter $\varepsilon$.

## Statement of the problem

We considered a SPFIDE with integral initial condition of the form:

1 $$\begin{aligned}L_{\varepsilon }u & :=\varepsilon u^\prime(x)+a(x)u(x)+\lambda \int _{0}^{l}K(x,s)u(s)ds=f(x),\quad x\in \Omega ,\\ u(0) &=\int _{0}^{l}c(s)u(s)ds+A, \end{aligned}$$

where $\Omega =(0,l]$, $({{\bar{\Omega }}}=\Omega \cup {x=0})$, $0<\varepsilon \ll 1$, is the perturbation parameter. $\lambda$ and *A* are given constants different from zero. We assumed that $a(x)\ge \alpha > 0$, $c(x)\ge 0$, *f*(*x*) and *K*(*x*, *s*) are sufficiently smooth functions satisfying certain regularity conditions. Under this condition, the solution *u*(*x*) of (1) exhibits a left boundary layer at $x=0$ for small value of $\varepsilon$. This means that the derivative of the solution becomes unbounded for small values of the perturbation parameter as *x* approaches to 0.

The following lemma establishes a prior estimate for the asymptotic behavior of the solution.

### Lemma 2.1

[9, 10] Let $a,f\in C(\Omega )$ and $K\in C(\Omega \times \Omega )$. Then, the solution of (1) holds:

2 $$\begin{aligned} \Vert u||_{\infty }\le C, \end{aligned}$$

where, $C=\left( B+\alpha ^{-1}||f||_{\infty }\right) e^{\frac{\lambda {\bar{K}}l}{\alpha }}, B=u(0)=\int _{0}^{l}u(s)c(s)ds+A$ and ${\bar{K}}=\max |K(x,s)|.$ In addition, if $\left| \frac{\partial }{\partial x}K(x,s)\right| \le {\bar{K}}_1<\infty$. Then, the solution satisfies,

3 $$\begin{aligned} |u^{(t)}(x)|\le c\left( 1+\frac{1}{\varepsilon ^t}e^{-\frac{\alpha x}{\varepsilon }}\right) ,\;x\in {\bar{\Omega }},\;\; t=0,1,2, \end{aligned}$$

where *c* is constant independent of $\varepsilon$.

## The fitted operator difference scheme

In this section, the exponentially fitted operator finite difference methods together with composite Simpson’s $\frac{1}{3}$ rule is applied to discretize the first-order SPFIDEs. On the domain [0, *l*], we introduce the equidistant meshes by dividing the domain using uniform mesh $h=x_{i}-x_{i-1}$ such that $0=x_0<x_1<x_2<...<x_N=l$ and where *N* is the discretization parameter. Using the Taylor series expansion for *u* about the point $x_i$, written as,

4 $$\begin{aligned}&u(x_{i-1})\approx u_{i-1}=u_i-hu^\prime_i + \frac{h^2}{2}u^{\prime\prime}_i +O(h^3), \end{aligned}$$

5 $$\begin{aligned}&u(x_{i+1})\approx u_{i+1}=u_i+hu^\prime_i + \frac{h^2}{2}u{\prime\prime}_i +O(h^3). \end{aligned}$$

Using (4) and (5), we obtain the following finite different operator,

6 $$\begin{aligned} \begin{array}{ll} D^- u_i = \frac{u_i -u_{i-1}}{h}+O(h), \;\; D^+ u_i = \frac{u_{i+1} -u_{i}}{h}+O(h), \;\; D^0 u_i = \frac{u_{i+1} -u_{i-1}}{2h}+O(h^2). \end{array} \end{aligned}$$

For the equation

7 $$\begin{aligned} \varepsilon u'(x) +a(x)u(x)=0, \end{aligned}$$

the fitting factor for the finite difference approximation of the equation can be computed as

8 $$\begin{aligned} \varepsilon \sigma \left( \frac{u_{i+1}-u_{i}}{h}\right) +\alpha u_{i} =0, \end{aligned}$$

where $\sigma$ is the introduced fitting factor. Let us denote $\rho$=$\frac{h}{\varepsilon }$, then by evaluating the limit of (8) as $h \rightarrow 0$,

9 $$\begin{aligned} \sigma = -\frac{\rho \alpha \lim \limits _{h\rightarrow 0} u_{i}}{\lim \limits _{h\rightarrow 0}\left( u_{i+1}-u_{i}\right) }. \end{aligned}$$

The asymptotic solution of (8), has the form

10 $$\begin{aligned} u(x)=u_0(x)+w(x), \end{aligned}$$

where $u_{0}(x)$ is the solution of the equation $\alpha u_{0}(x)=0$ and *w*(*x*) is the inner layer solution. Using (8) into (10), we have,

$$\begin{aligned} \begin{aligned} \varepsilon w'(x)+\alpha w(x) =0, \end{aligned} \end{aligned}$$

which gives $w(x)=Ce^{-\frac{\alpha x}{\varepsilon }}.$ Hence, solution of (10), re-written as, $u(x)=u_{0}(x)+Ce^{-\frac{\alpha x}{\varepsilon }}.$ But, from initial condition of (1) let, $u(0)=\int _{0}^{l}c(s)u(s)ds+A=B, \Rightarrow C=B-u_{0}(0).$ Therefore, the considered asymptotic solution is,

$$\begin{aligned} \begin{aligned} u(x)=u_0(x)+\left( B-u_0(0)\right) e^{-\frac{\alpha x}{\varepsilon }}. \end{aligned} \end{aligned}$$

Clearly, the stated first order singularly perturbed initial value problem has left boundary layers and on the uniform discretization point $x_{i}=ih$, we have,

11 $$\begin{aligned} \begin{aligned} \left. \begin{array}{ll} \lim \limits _{h\rightarrow 0} u_i = u_0(x)+\left( B-u_0(0)\right) e^{-\alpha i\rho }, \\ \lim \limits _{h\rightarrow 0} u_{i+1} = u_0(x)+\left( B-u_0(0)\right) e^{-\alpha (i+1)\rho }=u_0(x)+\left( B-u_0(0)\right) e^{-\alpha i\rho }e^{-\alpha \rho }, \\ \lim \limits _{h\rightarrow 0} u_{i-1} = u_0(x)+\left( B-u_0(0)\right) e^{-\alpha (i-1)\rho }=u_0(x)+\left( B-u_0(0)\right) e^{-\alpha i\rho }e^{\alpha \rho }. \end{array} \right\} \end{aligned} \end{aligned}$$

Using (11) into (9) and simplifying, the induced fitting parameter becomes,

$$\begin{aligned} \sigma =\frac{\rho \alpha }{1-e^{-\alpha \rho }}. \end{aligned}$$

By substituting the fitting factor $(\sigma )$ into the semi-discrete scheme we obtain,

12 $$\begin{aligned}L_{\varepsilon }^N u_i \equiv \big (\frac{\alpha }{1-e^{-\alpha \rho }}+a_i\big )u_{i} -\frac{\alpha }{1-e^{-\alpha \rho }} u_{i-1} + \lambda \int _{0}^{l}K(x_{i},s_{i})u(s_i)ds=f_i. \end{aligned}$$

Now, the truncation error of scheme (12) is given by,

$$\begin{aligned} L_{\varepsilon }^{N}(u(x_{i})-u_{i})=&\left( \epsilon u'(x_{i}) +a(x_{i}) u(x_{i})+\lambda \int _{0}^{l}K(x_{i},s_{i})u(s_i)\right) \\ {}&- \left( \big (\frac{\alpha }{1-e^{-\alpha \rho }}+a_i\big )u_{i} -\frac{\alpha }{1-e^{-\alpha \rho }} u_{i-1} + \lambda \int _{0}^{l}K(x_{i},s_{i})u(s_i)ds\right) \\ =&\varepsilon u'(x_{i})-\frac{\alpha }{1-e^{-\alpha \rho }}u_{i}+\frac{\alpha }{1-e^{-\alpha \rho }}u_{i-1}. \end{aligned}$$

Using the Taylor’s series in (6) we have,

13 $$\begin{aligned}\varepsilon u'(x_{i})-\frac{\alpha }{1-e^{-\alpha \rho }}u_{i}+\frac{\alpha }{1-e^{-\alpha \rho }}\left( u_{i}-hu^\prime_{i}+\frac{h^2}{2}u^{\prime\prime}_{i}(\xi )\right) =R_1, \end{aligned}$$

where $R_{1}$ is the truncated terms of the differential part of the problems. Moreover, applying the composite Simpson 1/3 rule to the integral term in (12), we form the complete discrete scheme. The Simpson’s 1/3 rule for approximating the integral part is given as

$$\begin{aligned} \int _{s_0}^{s_2}K(x_i,s)u(s)ds=\frac{h}{3}\left[ K(x_i,s_0)u(s_0)+4K(x_i,s_1)u(s_1)+K(x_i,s_2)\right] +\frac{-1}{90}h^5K_{i,0}u_0^{(iv)}(s). \end{aligned}$$

Generally, the composite Simpson’s 1/3 rule requires an even number of sub-divisions. Let [0, *l*] be sub-divided into *N* even number of sub-divisions, $0=s_0<s_1<s_2<\dots <s_N=l$, then the integral over the whole interval is found by adding this integration which yields,

14 $$\begin{aligned} &\int _{0}^{l}K(x_i,s)u(s)ds\\ {}&=\frac{h}{3}\left( K(x_i,s_0)u(s_0)+4\sum _{j=1}^{N/2}K(x_i,s_{2j-1})u(s_{2j-1})+2\sum _{j=1}^{N/2-1}K(x_i,s_{2j})u(s_{2j})+K(x_i,s_N)u(s_N)\right) +R_2. \end{aligned}$$

where, $R_2=\frac{-1}{90}h^5\left[ K_{i,0}u_0^{(iv)}(\xi )+K_{i,2}u_2^{(iv)}(\xi )+\dots +K_{i,N-2}u_{N-2}^{(iv)}(\xi )\right] =\frac{-l}{180}h^4u^{(iv)}(\xi ),\ \xi \in [0,l].$ where $u^{(iv)}(\xi )$ is the largest value of the *N*- quantities on the $4^{th}$ derivatives. Clearly, from (12) and (14) we have the following exact relation for $u(x_i),$

15 $$\begin{aligned} L_\epsilon ^Nu_i:= \bigg (\frac{\alpha }{1-e^{-\alpha \rho }}+a_{i}\bigg )u_{i}- \frac{\alpha }{1-e^{-\alpha \rho }} u_{i-1} +\lambda h\sum _{j=0}^{N}\eta _jK_{ij}u_j+R=f_i, \;\; 1\le i\le N-1, \end{aligned}$$

where

$$\begin{aligned} \eta _j = \left\{ \begin{array}{ll} \frac{1}{3},&{} \hbox { for}\ j=0, N , \\ \frac{4}{3},&{} \hbox { for}\ j= 1, 3, 5, \dots , N-1 , \\ \frac{2}{3}, &{} \hbox { for}\ j= 2, 4, 6, \dots , N-2 , \end{array} \right. \end{aligned}$$

where $R=R_1+R_2$. Similar to the approaches for an integral part of the problem (14) the initial condition of (1) is discretized as,

16 $$\begin{aligned} u(0)=\frac{h}{3}\left( c(x_0)u(x_0)+2\sum _{i=1}^{\frac{N}{2}-1}c(x_{2i})u(x_{2i})+4\sum _{i=1}^{\frac{N}{2}}c(x_{2i-1})u(x_{2i-1})+c(x_N)u(x_N)\right) +A+R_3. \end{aligned}$$

In a similar manner, we obtain the errors in the remaining sub-interval becomes, $R_3=\frac{-1}{180}h^{4}\left( c^{4}(\xi )u^{4}(\xi )\right) .$ Based on (15) and (16) we propose the following difference scheme for approximating the problem (1)

17 $$\begin{aligned} L_\varepsilon ^Nu_i:=&\bigg (\frac{\alpha }{1-e^{-\alpha \rho }}+a_{i}\bigg )u_{i}- \frac{\alpha }{1-e^{-\alpha \rho }} u_{i-1} +\lambda h\sum _{j=0}^{N}\eta _jK_{ij}u_j=f_i, \\&u(0)=h\sum _{i=0}^{N}\eta _ic_{i}u_i+A, \end{aligned}$$

where

$$\begin{aligned} \eta _j = \left\{ \begin{array}{ll} \frac{1}{3},&{} \hbox { for}\ j=0, N , \\ \frac{4}{3},&{} \hbox { for}\ j= 1, 3, 5, \dots , N-1 , \\ \frac{2}{3}, &{} \hbox { for}\ j= 2, 4, 6, \dots , N-2 . \end{array} \right. \end{aligned}$$

Lastly, from (17) the linear system of equations for $u_1, u_2, u_3, \dots , u_{N-1}$ are generated. Therefore, the generated system of linear algebraic equations can be written in matrix form as,

$$\begin{aligned} (M + T)u=F, \end{aligned}$$

where *M* and *T* are coefficient matrix, *F* is a given function and *u* is an unknown function that is to be determined. The entries of *M*, *T* and *F* are given as,

$$\begin{aligned} M = \left\{ \begin{array}{ll} m_{11}=&{}\frac{\alpha }{1-e^{-\alpha \rho }}+a_{i}+h\eta _{1}c_{1}+A,\\ m_{1i}=&{}h\eta _{i}c_{i}u_{i} +A, \; \hbox { for}\ i=2,3,\ldots ,N-1,\\ m_{ii}=&{} \frac{\alpha }{1-e^{-\alpha \rho }}+\alpha ,\; \hbox { for}\ i=1, \dots , N , \\ m_{ii-1}=&{} \frac{- \alpha }{1-e^{-\alpha \rho }}, \; \hbox { for}\ i= 1, 2, \dots , N-1 ,\\ \end{array} \right. \\ T= \left\{ \begin{array}{ll} \frac{4h\lambda }{3}K_{i,2j-1},\; \hbox { for}\ j=1, 2, \dots ,\frac{N}{2} ,\\ \frac{2h\lambda }{3}K_{i,2j},\; \hbox { for}\ j=1, 2, \dots , \frac{N}{2}-1 , \end{array} \right. \end{aligned}$$

and

$$\begin{aligned}F= \left\{ \begin{array}{l} f_{1} + \frac{\alpha }{1-e^{-\alpha \rho }}\left( h\eta _{0}c_{0}u_{0}+A\right) +\lambda h\left( \eta _{0}K_{1,0}+\eta _{N}K_{1,N}\right) ,\\ f_{i}-\lambda h\left( \eta _0K_{i,0}+\eta _N K_{i,N}\right) , \hbox { for \;}\ i= 2, 3, \dots , N-2. \end{array} \right. \end{aligned}$$

### Stability and uniform convergence analysis

In this section, we need to show the discrete scheme in (17) satisfies the discrete maximum principle, uniform stability estimates, and uniform convergence.

#### Lemma 3.1

(Discrete maximum principle). Assume that $\lambda h\sum _{j=0}^{N}\eta _j K_{ij}y_j \le \alpha .$ Let the difference operator,

$$\begin{aligned}L_\epsilon ^Ny_i= \bigg (\frac{\alpha }{1-e^{-\alpha \rho }}+a_i \bigg ) y_{i}- \frac{\alpha }{1-e^{-\alpha \rho }} y_{i-1} +\lambda h\sum _{j=0}^{N}\eta _j K_{ij}y_j, 1\le i \le N, \end{aligned}$$

be given. Then, for all mesh function $y_{i}$ such that $y_{0} \ge 0$. Then $L_{\varepsilon }^{N} y_{i} \ge 0$, implies that $y_{i} \ge 0,$ for all $0 \le i \le N$.

#### Proof

Let *m* be such that $y_{m} = \min _{0\le i\le N} y_{i}$ and suppose that $y_{m} <0$. It is clear that, $m \ne 0$, $y_{m} \le y_{m+1}$ and $y_{m} \le y_{m-1}$. If $\sigma =\frac{\alpha \rho }{1-e^{-\alpha \rho }}$ and $\rho =\frac{h}{\varepsilon }$, it follows that,

$$\begin{aligned} &L_\varepsilon ^N y_{m}= \bigg (\frac{\alpha }{1-e^{-\alpha \rho }}+a_m \bigg ) y_{m}- \frac{\alpha }{1-e^{-\alpha \rho }} y_{m-1} +\lambda h\eta _m K_{im}y_{m},\\&=\bigg (\frac{\alpha }{1-e^{-\alpha \rho }}+a_m +\lambda h\eta _m K_{im}\bigg )y_{m}-\frac{\alpha }{1-e^{-\alpha \rho }}y_{m-1}< 0, as \alpha >0, \end{aligned}$$

which is contradiction with the assumption that it made above $L_{\varepsilon }^{N} y_{i} \ge 0$. It follows that $y_{m}>0$ and thus $y_{i}>0$, $\forall i, 0\le i\le N$. $\square$

The uniqueness of the solution is guaranteed by this discrete maximum principle. The existence follows easily since, as for linear problems, the existence of the solution is implied by its uniqueness [9]. The discrete maximum principle enables us to prove the following lemma which provides the boundedness of the solution.

#### Lemma 3.2

Let linear difference operator $L_{\varepsilon }^Ny_i$ be defined as,

$$\begin{aligned} L_\epsilon ^Ny_i= \bigg (\frac{\alpha }{1-e^{-\alpha \rho }}+a_i \bigg ) y_{i}- \frac{\alpha }{1-e^{-\alpha \rho }} y_{i-1} +\lambda h\sum _{j=0}^{N}\eta _j K_{ij}y_j, 1\le i \le N, \end{aligned}$$

then, the following inequality holds,

$$\begin{aligned}\Vert y\Vert _{\infty } \le \Vert L_\varepsilon ^Ny\Vert _{\infty }. \end{aligned}$$

#### Proof

Consider the function $\varphi ^{\pm }$ defined by, $\varphi ^{\pm }_{i}= \Vert L_\varepsilon ^Ny\Vert _{\infty ,\Omega _N} \pm y_{i}$. At initial condition, $x=0$ we have,

$$\begin{aligned} \varphi ^{\pm }_{0} = \Vert L_\varepsilon ^Ny\Vert _{\infty ,\Omega _N}\pm y_{0}=\Vert f\Vert _{\infty ,\Omega _N}\pm y_{0}\ge 0. \end{aligned}$$

For $x>0$ we have,

$$\begin{aligned} \varphi ^{\pm }(x_i)= \Vert L_\varepsilon ^Ny\Vert _{\infty ,\Omega _N}\pm y(x_i)=\Vert L_\varepsilon ^Ny\Vert _{\infty ,\Omega _N}\pm y(x_i)\ge 0. \end{aligned}$$

This implies $\varphi ^{\pm }_i \ge 0$, $\forall x_{i}\in [0,l]$. Therefore, from Lemma 3.2 it follows that, $|y(x_i)|\le \Vert L_\varepsilon ^Ny\Vert _{\infty ,\Omega _N}, \forall x_{i}\in \Omega _{N},$ which completes the proof. $\square$

#### Theorem 3.1

If $a,\ f \in C({\bar{\Omega }})$, $\frac{\partial ^s K}{\partial x^s}\in C({\bar{\Omega }}\times {\bar{\Omega }})$, $s=0,1$ and $|\lambda |<\frac{\alpha }{\max _{1\le i \le N}\sum _{j=0}^{N}h\eta _j|K_{ij}|},$ then, the solution of (17) converges uniformly to the solution of (1). The error of the approximated solution of (1) satisfies the bound

$$\begin{aligned} \Vert u(x_i)-u_{i}\Vert _{\infty ,{\bar{\Omega }}_N}\le Ch. \end{aligned}$$

#### Proof

Suppose the error of the approximate solution is given by $z_i:=u(x_i)-u_i$ then from (17) we have,

$$\begin{aligned} &L_\epsilon ^Nz_i= \bigg (\frac{\alpha }{1-e^{-\alpha \rho }}+a_i\bigg )z_{i}- \frac{\alpha }{1-e^{-\alpha \rho }} z_{i-1} +\lambda h\sum _{j=0}^{N}\eta _j K_{ij}z_j=R,\\&z_0=h\sum _{i=0}^{N}\eta _ic_{i}z_i+u(0), where\, R=R_{1}+R_{2}\, and\, 1\le i\le N. \end{aligned}$$

Using the result in Lemma 3.2 we have

$$\begin{aligned} \Vert z\Vert _{\infty ,{\bar{\Omega }}_N}\le C\Vert R\Vert _{\infty ,\Omega _N}. \end{aligned}$$

However we estimate the error *R* from (13) and (3)

$$\begin{aligned} |R|=|R_{1}+R_{2}| =&\left| \varepsilon u'(x_{i})+a_i u(x_{i})-\bigg (\frac{\alpha }{1-e^{-\alpha \rho }}+a_i\bigg )u_{i}+\frac{\alpha }{1-e^{-\alpha \rho }}\left( u_{i}-hu'_{i}+\frac{h^2}{2}u''_{i}(\xi )\right) +\frac{h^4}{24}u^{(iv)}(\xi )\right| \\ =&\left| \frac{h}{\rho } u'_{i}-\frac{\alpha }{1-e^{-\alpha \rho }}\left( hu'_{i}+\frac{h^2}{2}u''_{i}(\xi )\right) +\frac{h^4}{24}u^{(iv)}(\xi )\right| \\ \le&\left| \left( \frac{1}{\rho }-\frac{\alpha }{1-e^{-\alpha \rho }}\right) hu'_{i}\right| +\left| \frac{\alpha }{2(1-e^{-\alpha \rho })}{h^2}u''_{i}\right| +\left| \frac{1}{24}h^{4}u^{(iv)}(\xi )\right| . \end{aligned}$$

Using the bounds of $t^{th}$ derivatives of the solution of the problem in (3) we have

$$\begin{aligned} R \le&\left| \left( \frac{1}{\rho }-\frac{\alpha }{1-e^{-\alpha \rho }}\right) \left( 1+\frac{1}{\varepsilon }e^{\frac{-\alpha x_i}{\varepsilon }}\right) \right| h+\left| \frac{\alpha }{2(1-e^{-\alpha \rho })}\left( 1+\frac{1}{\varepsilon ^2}e^{\frac{-\alpha x_i}{\varepsilon }}\right) \right| h^2+\left| \frac{1}{24}\left( 1+\frac{1}{\varepsilon ^4}e^{\frac{-\alpha x_i}{\varepsilon }}\right) \right| h^{4}. \end{aligned}$$

We have that $h^4\le h^2\le h$ and from the Taylor series approximation of $1-e^{-\alpha \rho }=\alpha \frac{h}{\varepsilon }-\alpha \frac{h^2}{\varepsilon ^2}+...$, the estimated error can be written as, $R\le Ch,$ which gives

18 $$\begin{aligned} \Vert R\Vert _{\infty ,\Omega _N}\le Ch. \end{aligned}$$

Hence, from the bound in (18) the proposed numerical solution is uniformly convergent to the analytical solution.$\square$

## Numerical examples and discussions

To verify the feasibility of the established theoretical results in this paper, we consider the experiments of two specific examples using the proposed numerical scheme on the problem of the form given in (1). We use the double mesh principle to estimate the maximum absolute errors.

### ***Example 4.1***

Consider the singularly perturbed problem from [9]

$$\begin{aligned} Lu:=&\varepsilon u'(x)+u(x)+\frac{1}{20}\int _{0}^{1}xu(s)ds\\=&-\frac{\varepsilon }{(1+x)^2}+\frac{1}{1+x}+x\varepsilon (1-e^{-\frac{x}{\varepsilon }})+x\ln (1+x)-\frac{19}{20}x\left[ \varepsilon \left( 1-e^{-\frac{x}{\varepsilon }}\right) +\ln (1+x)\right] \\ {}&+\frac{1}{20}x\left[ \varepsilon \left( e^{-\frac{x}{\varepsilon }}-e^{-\frac{1}{\varepsilon }}\right) +\ln \left( \frac{2}{1+x}\right) \right] , ~~~0<x\le 1, \end{aligned}$$

with the initial condition

$$\begin{aligned} u(0)=-\int _{0}^{1}su(s)ds+4+\varepsilon ^2+(2-\varepsilon (1+\varepsilon ))e^{-\frac{1}{\varepsilon }}-\ln 2. \end{aligned}$$

**Fig. 1:**
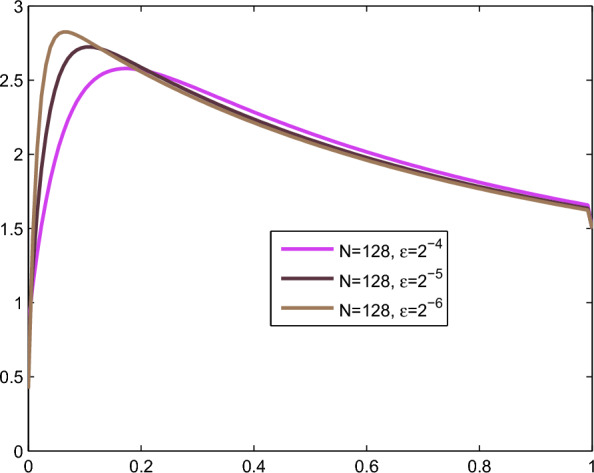
Numerical solution of Example 4.1 with boundary layer formation as $\varepsilon =2^{-4},2^{-5}$ and $2^{-6}$ goes small with the corresponding mesh number $N=128$

### ***Example 4.2***

Consider the singularly perturbed problem from [9]

$$\begin{aligned} Lu:=\varepsilon u'(x)+2u(x)+\frac{1}{10}\int _{0}^{1}e^{1-xs}u(s)ds=2x+1,~~~~0<x\le 1, \end{aligned}$$

with the initial condition

$$\begin{aligned} u(0)+\int _{0}^{1}sin\left( \frac{\pi s}{2}\right) u(s)ds=-2. \end{aligned}$$

**Fig. 2:**
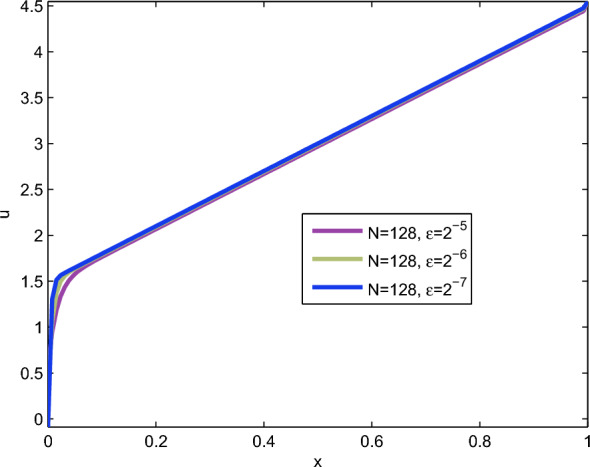
Numerical solution of Example 4.2 with boundary layer formation as $\varepsilon =2^{-5},2^{-6}$ and $2^{-7}$ goes small with corresponding mesh number $N=128$

**Fig. 3:**
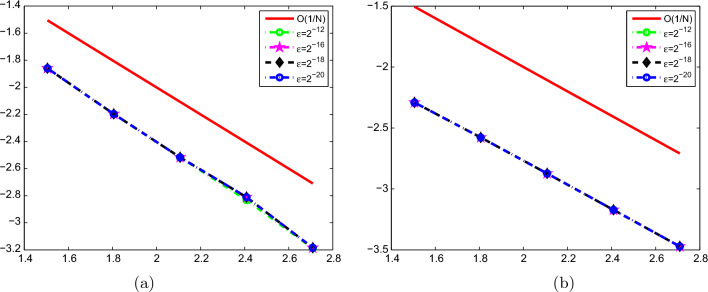
Log-Log plot of the maximum absolute error for different values of the perturbation parameter and mesh numbers: **a** Example 4.1 and **b** Example 4.2

Since the exact solution to both of the examples are unknown, we estimate the maximum absolute errors and calculate the solutions using the double-grid method, in which the solution obtained is compared with the solution calculated on a double-fine grid. We denote the maximum absolute errors using the formula

$$\begin{aligned} E_{\varepsilon }^{N}=\max _{0\le i\le N}|u^{N}_{i}-u^{2N}_{2i}|, \end{aligned}$$

where $u^{N}_{i}$ is the computed solution on *N* number of mesh points and $u^{2N}_{2i}$ is the computed solution on 2*N* number of of mesh points. The rate of convergence is calculated by the formula

$$\begin{aligned} R^{N}=\log _{2}\left( \frac{E_{\varepsilon }^{N}}{E_{\varepsilon }^{2N}}\right) . \end{aligned}$$

**Table 1:** Maximum absolute error and rate of convergence of the proposed method for Example 4.1

$\varepsilon \downarrow N \rightarrow$	$2^5$	$2^6$	$2^7$	$2^8$	$2^9$	$2^{10}$
$2^{0}$	1.3116e–02	6.9097e–03	3.5442e–03	1.7946e–03	9.0298e–04	4.5291e–04
	0.9246e+00	0.9631e+00	0.9817e+00	0.9908e+00	0.9954e+00	
$2^{-4}$	1.3387e–02	7.4664e–03	3.9318e–03	2.0162e–03	1.0207e–03	5.1355e–04
	0.8424e+00	0.9252e+00	0.9635e+00	0.9820e+00	0.9909e+00	
$2^{-8}$	1.7334e–03	2.3379e–03	3.9174e–03	5.2115e–03	5.1020e–03	3.7144e–03
	0.4316e+00	0.7446e+00	0.4118e+00	0.0306e+00	0.4590e+00	
$2^{-12}$	1.9236e–03	9.0871e–04	3.9685e–04	7.3668e–04	2.1274e–03	4.4521e–03
	1.0819e+00	1.1952e+00	0.8924e+00	1.5299e+00	1.0653e+00	
$2^{-16}$	1.9236e–03	9.0871e–04	3.9685e–04	7.3668e–04	2.1274e–03	4.4521e–03
	1.0819e+00	1.1952e+00	0.8924e+00	1.5299e+00	1.0653e+00	
$2^{-18}$	1.9236e–03	9.0871e–04	3.9685e–04	7.3668e–04	2.1274e–03	4.4521e–03
	1.0819e+00	1.1952e+00	0.8924e+00	1.5299e+00	1.0653e+00	
$2^{-20}$	1.9236e–03	9.0871e–04	3.9685e–04	7.3668e–04	2.1274e–03	4.4521e–03
	1.0819e+00	1.1952e+00	0.8924e+00	1.5299e+00	1.0653e+00

**Table 2:** Comparison of the maximum absolute error of the proposed scheme and the scheme in [9] of Example 4.1

$\varepsilon \downarrow N \rightarrow$	$2^6$	$2^7$	$2^8$	$2^9$
Proposed scheme				
$2^{-4}$	7.4664e–03	3.9318e–03	2.0162e–03	1.0207e–03
$2^{-8}$	2.3379e–03	3.9174e–03	5.2115e–03	5.1020e–03
$2^{-10}$	9.0871e–04	3.9685e–04	7.3668e–04	2.1274e–03
Result in [9]				
$2^{-4}$	4.358e–01	5.281e–01	6.133e–01	5.291e–01
$2^{-8}$	4.912e–01	4.830e–01	5.668e–01	6.435e–01
$2^{-10}$	7.700e–01	4.610e–01	5.701e–01	6.014e–01

**Table 3:** Maximum absolute error and rate of convergence of the proposed method for Example 4.2

$\varepsilon \downarrow N \rightarrow$	$2^5$	$2^6$	$2^7$	$2^8$	$2^9$	$2^{10}$
$2^{0}$	3.4992e–02	1.8983e–02	9.8939e–03	5.0518e–03	2.5526e–03	1.2831e–03
	0.8823e+00	0.9400e+00	0.9697e+00	0.9848e+00	0.9923e+00	
$2^{-4}$	1.3018e–01	1.3971e–01	1.0548e–01	6.6102e–02	3.7290e–02	2.9853e–03
	0.1019e+00	0.4054e+00	0.6742e+00	0.8259e+00	0.9094e+00	
$2^{-8}$	7.7364e–03	4.3025e–03	6.3198e–03	6.3217e–03	1.3572e–03	1.4234e–03
	0.8455e+00	0.5547e+00	0.7891e+00	0.8023e+00	0.9821e+00	
$2^{-12}$	7.7206e–03	3.8803e–03	1.9451e–03	9.7376e–04	4.8817e–04	2.7002e–04
	0.9925e+00	0.9963e+00	0.9982e+00	0.9960e+00	0.9981e+00	
$2^{-16}$	7.7206e–03	3.8803e–03	1.9451e–03	9.7376e–04	4.8718e–04	2.4367e–04
	0.9925e+00	0.9963e+00	0.9982e+00	0.9991e+00	0.9995e+00	
$2^{-18}$	7.7206e–03	3.8803e–03	1.9451e–03	9.7376e–04	4.8718e–04	2.4367e–04
	0.9992e+00	0.9963e+00	0.9982e+00	0.9991e+00	0.9995e+00	
$2^{-20}$	7.7206e–03	3.8803e–03	1.9451e–03	9.7376e–04	4.8718e–04	2.4367e–04
	0.9992e+00	0.9963e+00	0.9982e+00	0.9991e+00	0.9995e+00

**Table 4:** Comparison of the maximum absolute error of the proposed scheme and the scheme in [9] of Example 4.2

$\varepsilon \downarrow N \rightarrow$	$2^6$	$2^7$	$2^8$	$2^9$	$2^{10}$
Proposed scheme					
$2^{-4}$	1.8983e−02	1.0548e−02	6.6102e–02	3.7290e–02	2.9853e–03
$2^{-8}$	4.3025e−03	6.3198e−03	6.3217e–03	1.3572e–03	1.4234e–03
$2^{-12}$	3.8803e−03	1.9451e−03	9.7376e–04	4.8718e–04	2.4367e–04
$2^{-16}$	3.8803e−03	1.9451e–03	9.7376e–04	4.8718e–04	2.4367e–04
Result in [9]					
$2^{-4}$	5.558e–02	1.687e−02	4.810e–02	1.270e–03	3.200e–04
$2^{-8}$	5.610e–02	1.703e–02	4.960e–03	1.320e–03	3.400e–04
$2^{-12}$	5.544e–02	1.683e–02	4.970e–03	1.350e–03	3.500e–04
$2^{-16}$	5.680e–02	1.736e–02	5.160e–03	1.420e–03	3.700e–04

We computed the maximum absolute error and the rate of convergence for the given examples by varying the number of mesh *N* and the perturbation parameter $\varepsilon$. In Table 1 and Table 3, the maximum absolute error and rate of convergence of the proposed scheme is presented. The findings, indicates that as the perturbation parameter decreases, the developed scheme achieves a stable and bounded maximum absolute error. This suggests that, the maximum absolute error of the scheme remains unaffected by the perturbation parameter $\varepsilon$, which indicates the uniform convergence. By examining the values of $\varepsilon$ and *N* in both Example 4.1 and Example 4.2, we observe the uniform convergence and the rate of convergence steadily increase towards unity, confirming the assertion made in Theorem 3.1.

To observe the behaviour of the considered problem, we plot the numerical solution profiles for various small values of the perturbation parameter $\varepsilon$ in Figure 1 and Figure 2. Furthermore, in Tables 2 and 4, we compare the maximum absolute error of the proposed scheme with that of the scheme in [9] for both of the considered examples. The tables demonstrate that the proposed scheme outperforms the result in [9] in terms of the maximum absolute error.

## Conclusions

We have constructed a fitted operator finite difference scheme, together with the composite Simpson’s $\frac{1}{3}$ rule for the problem on a uniform mesh, to provide a numerical solution of the first-order singularly perturbed Fredholm integro differential equation with the boundary layer. We have shown that the method is uniformly first-order convergent concerning the perturbation parameter $\varepsilon$. As it can be seen in Table 1 and Table 3, and the Log-Log plot in Figure 3, the numerical results of the test problems also agree with the analysis of the error estimates and with the order of convergence and hence, it is confirmed that the convergence order of the scheme is $O(N^{-1})$ where *N* is the number of mesh intervals. The influence of the perturbation parameter on solving the problem is shown in the figures. The effectiveness of the proposed scheme is verified by comparing the results with previous studies. The proposed method was found to provide more accurate and stable numerical results. This study focused on the application of the fitted operator finite difference method in conjunction with Simpson’s $\frac{1}{3}$ rule to solve linear first-order singularly perturbed Fredholm’s integro-differential equations on a uniform grid. The fitted finite difference method is very applicable to get stable numerical solutions to singularly perturbation problems. Therefore, we suggest that in future work, researchers could extend the fitted finite difference method with the corresponding quadrature rule to solve linear singularly perturbed problems with delay.

## Data Availability

The original contributions presented in the study are included in the article/supplementary materials, further inquiries can be directed to the corresponding author.

## References

[1] Amirali I, Durmaz M, Acar H, Amiraliyev G (2023). First-order numerical method for the singularly perturbed nonlinear fredholm integro-differential equation with integral boundary condition. J Phys Conf Ser.

[2] Amiraliyev GM, Durmaz ME, Kudu M (2018). Uniform convergence results for singularly perturbed fredholm integro-differential equation. J Math Anal..

[3] Amiraliyev GM, Durmaz ME, Kudu M (2020). Fitted second order numerical method for a singularly perturbed fredholm integro-differential equation. Bull Belgian Math Soc Simon Stevin.

[4] Badeye SR, Woldaregay MM, Dinka TG (2023). Solving singularly perturbed fredholm integro-differential equation using exact finite difference method. BMC Res Notes.

[5] Cakir M, Ekinci Y, Cimen E (2022). A numerical approach for solving nonlinear fredholm integro-differential equation with boundary layer. Comput Appl Math.

[6] Cakir M, Gunes B (2022). A fitted operator finite difference approximation for singularly perturbed volterra-fredholm integro-differential equations. Mathematics.

[7] Durmaz ME (2023). A numerical approach for singularly perturbed reaction diffusion type volterra-fredholm integro-differential equations. J Appl Math Comput.

[8] Durmaz ME, Amiral G, Kudu M (2022). Numerical solution of a singularly perturbed fredholm integro differential equation with robin boundary condition. Turk J Math.

[9] Durmaz ME, Amirali I, Amiraliyev GM (2023). An efficient numerical method for a singularly perturbed fredholm integro-differential equation with integral boundary condition. J Appl Math Comput.

[10] Durmaz ME, Amirali I, Mohapatra J, Amiraliyev GM (2023). A second-order numerical approximation of a singularly perturbed nonlinear fredholm integro-differential equation. Applied Numerical Mathematics.

[11] Durmaz ME, Amiraliyev GM (2021). A robust numerical method for a singularly perturbed fredholm integro-differential equation. Mediterr J Math.

[12] Durmaz ME, Çakir M, Amiral G (2022). Parameter uniform second-order numerical approximation for the integro-differential equations involving boundary layers. Commun Facul Sci Univ Ankara Ser A1 Math Stat.

[13] Durmaz ME, Cakir M, Amirali I, Amiraliyev GM (2022). Numerical solution of singularly perturbed fredholm integro-differential equations by homogeneous second order difference method. J Comput Appl Math.

[14] Durmaz ME, Cakir M, M AG. Second order numerical method for the singularly perturbed fredholm integro-differential problem with zeroth order reduced equation. 2020.

[15] Durmaz ME, Yapman Ö, Mustafa K, Amiral G (2023). An efficient numerical method for a singularly perturbed volterra-fredholm integro-differential equation. Hacettepe J Math Stat.

[16] Hamoud AA, Ghadle KP. Modified variational iteration method for solving caputo fractional volterra-fredholm integro-differential equations. Int J Math Comput 2019;30(2).

[17] Hamoud AA, Ghadle KP (2019). Usage of the variational iteration technique for solving fredholm integro-differential equations. J Comput Appl Mech.

[18] Kudu M, Amirali G, Amiraliyev G (2018). Uniform numerical approximation for parameter dependent singularly perturbed problem with integral boundary condition. Miskolc Math Notes.

[19] Kudu M, Amirali I, Amiraliyev GM (2016). A finite-difference method for a singularly perturbed delay integro-differential equation. Journal of Computational and Applied Mathematics.

[20] Miller J, O’Riordan E, Shishkin G, Kellogg RB (1997). Fitted numerical methods for singular perturbation problems. SIAM Review.

[21] Nefedov NN, Nikitin AG (2007). The cauchy problem for a singularly perturbed integro-differential fredholm equation. Computational Mathematics and Mathematical Physics.

[22] Panda A, Mohapatra J (2023). A robust finite difference method for the solutions of singularly perturbed fredholm integro-differential equations. Mediterr J Math.

[23] Rathore AS, Shanthi V, Ramos H (2023). A fitted numerical method for a singularly perturbed fredholm integro-differential equation with discontinuous source term. Appl Num Math.

[24] Roos H, Stynes M, Tobiska L (1996). Numerical methods for singularly perturbed differential equations.

[25] Salama A, Evans DJ (2001). Fourth order scheme of exponential type for singularly perturbed volterra integro-differential equations. Int J Comp Math.

[26] Seal A, Natesan S (2023). Convergence analysis of a second-order scheme for fractional differential equation with integral boundary conditions. J Appl Math Comput.

[27] Turuna DA, Woldaregay MM, Duressa GF (2020). Uniformly convergent numerical method for singularly perturbed convection-diffusion problems. Kyungpook Math J.

